# Dimension, Location and Clinical Importance of Supraorbital Foramen and Notch: A Combined Cadaveric and Dry Skull Study

**DOI:** 10.1007/s00266-024-04196-7

**Published:** 2024-07-01

**Authors:** Ayşe Gül Kabakcı, Memduha Gülhal Bozkır

**Affiliations:** Faculty of Medicine Department of Anatomy, Cukurova University, Adana, Turkey

**Keywords:** Anatomy, Aesthetic, Cadaver, Dry skull, Supraorbital foramen, Supraorbital notch

## Abstract

**Purpose:**

The exit points of the supraorbital nerve and its branches hold significant importance in various medical procedures, including supraorbital endoscopic surgeries, forehead–facial aesthetic plastic surgeries, medical aesthetic applications and maxillofacial surgeries. Therefore, the primary objective of the present study was to precisely define the dimension, location and clinical significance of the supraorbital foramen/notch. By doing so, we aimed to enhance our understanding of this anatomical structure and its implications for surgical and aesthetic interventions in the supraorbital region.

**Methods:**

For our study, we conducted anatomical dissections and bone measurements to assess the significance of anatomical variations of the supraorbital foramen/notch. We utilized a sample consisting of 28 cadavers and 38 skulls. The supraorbital foramen/notch was bilaterally analyzed in all 28 cadavers and 38 dry bones. We morphometrically analyzed the distance between the supraorbital foramen/notch and various anatomical landmarks, including the nasion, supraorbital margin, infraorbital margin, temporal crest, glabella, frontal cavity and midline of the face. Additionally, we measured the distance between the supraorbital foramen/notches and the frontal foramen/notches, and the width of the supraorbital foramen/notch and the distance between both supraorbital foramina/notches.

**Results:**

There are 32 (57.14%) supraorbital  foramina, and the remaining are 24 (42.86%) supraorbital notches in cadavers and there are 36 (47.37%) supraorbital foramina, and the remaining are 40 (52.63%) supraorbital notches in skulls. We observed consistency in the dimension and location values of anatomical measurement parameters between cadavers and dry skulls on both right and left sides, with the exception of the parameter "distance from temporal crest" (*p*=0.042). Furthermore, our correlation analysis revealed a significant positive relationship between the right and left sides across all parameters, except for the following instances: in dry skulls, "distance from supraorbital margin" and in cadaver parameters, "distance from temporal crest, " "distance from frontal cavity" and "width."

**Conclusion:**

In our study, we observed that the distributions of supraorbital foramina and notches were nearly similar. Furthermore, our findings indicated comparable measurements between the right and left sides in both cadavers and skulls. These results suggest a degree of consistency in supraorbital anatomy within our study sample, regardless of the specimen type (cadavers or skulls) or laterality (right or left side).

**No Level Assigned:**

This journal requires that authors assign a level of evidence to each submission to which Evidence-Based Medicine rankings are applicable. This excludes Review Articles, Book Reviews, and manuscripts that concern Basic Science, Animal Studies, Cadaver Studies, and Experimental Studies. For a full description of these Evidence-Based Medicine ratings, please refer to the Table of Contents or the online Instructions to Authors www.springer.com/00266

## Introduction

The supraorbital margin is formed entirely by the squamous part of the frontal bone, which is interrupted at the junction of its sharp lateral 2/3rd and rounded medial 1/3rd by the supraorbital foramen/notch. Also, supraorbital vessels and nerve pass through it [1]. Significant anatomical variations associated with the morphology, occurrence and location of supraorbital nerve exits from the frontal bone have been reported [2]. The relationship between different skeletal landmarks can be used to create surgical guides. Thus, knowing where these landmarks is critical for surgeons to reduce the risk of injury to the vital nerves, arteries, and veins that cross them [3].

An ideal forehead shape should be full, with optimum convexity, and smoothly continue with the eyebrows, glabella, temples and then the cheekbones. Complete treatment of the upper face includes the forehead, glabella, eyebrows and temples. However, most physicians hesitate to perform procedures on the forehead and glabella area, mostly due to the dangerous vessels and nerves in this area. Knowing the anatomy of the facial area well forms the basis of aesthetic procedures and approaches to this area. This anatomical knowledge, including blood supply, nerve location and facial compartments, is essential in providing patients with the best treatment options, appropriately managing complications, achieving ideal results and avoiding undesirable side effects. Understanding the variations of supraorbital foramina in maxillofacial surgery is crucial for ensuring precision, reducing morbidity, facilitating surgical intervention and achieving more satisfactory outcomes. Previous studies utilizing cadavers, skulls and CT scans have reported anatomical variations in this region, which are particularly significant in oculoplastic and aesthetic surgery [4].

Cosmetic procedures targeting the glabella and supraorbital region necessitate a nuanced understanding of the intricate anatomical structures to mitigate potential complications. Various injectable substances, such as calcium hydroxyapatite and hyaluronic acid, are utilized in these procedures, necessitating precise injection techniques to ensure safety and efficacy. Furthermore, the significance of accurate localization of anatomical landmarks, particularly the supraorbital foramen/notch, is highlighted, with variations in these landmarks posing implications for both aesthetic interventions and underlying pathologies [5–7].

Therefore, in this study, we aimed to emphasize its clinical importance with morphometric measurements of the supraorbital foramen/notch on cadavers and dry skulls. The measurements in our study were taken on cadavers and dry bones used for education at the Anatomy Department of  Cukurova University Faculty of Medicine.

## Materials and Methods

Twenty–eight cadavers and thirty–eight dry skulls from our anatomical collection in Faculty of Medicine of Cukurova University were studied. Necessary permissions for the study were obtained from Cukurova University Medical Faculty, Non–invasive Clinical Research Ethic Board with conclusion number 142/10. The experimental procedures were conducted in accordance with the Declaration of Helsinki.

For this study, cadaver dissection was performed in the dissection room of the Anatomy Department. A total of twenty–eight frozen fresh head and neck cadavers in the anatomy department were dissected. Before dissection, the cadavers were removed from the freezer and allowed to thaw. After thawing, dissection was performed from frontal and orbital lines in each cadaver. First, dissection started from the lines marked in Figure 1.1 and continued until the orbital rim. In each cadaver, first the right side and then the left side were dissected (Figure 1.2). The supraorbital foramen, or notch, was found by tracing the arteria/vena/nervus supraorbitale and was dissected until it became fully evident (Figure 1.3, 1.4). Then, dissection was continued from supraorbital margo to infraorbital margo (Figure 2).

**Fig. 1:**
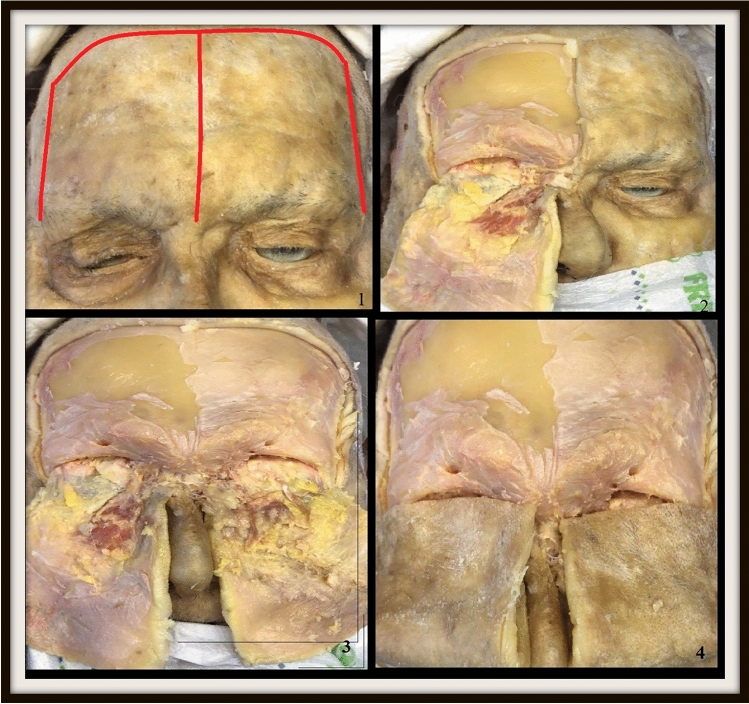
Some of the stages of dissection to reach the supraorbital foramen/notch

**Fig. 2:**
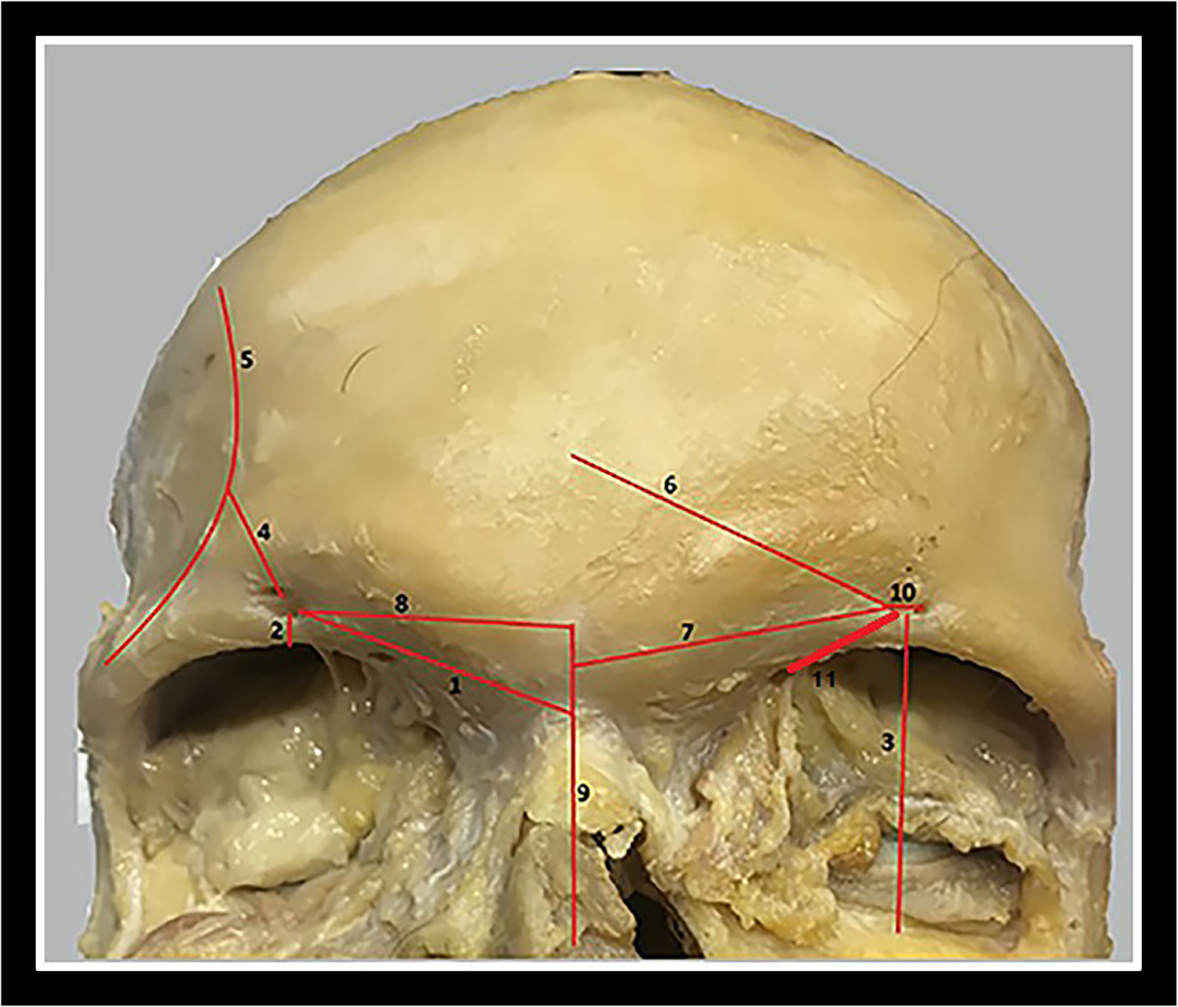
Landmarks of anatomical measurements on the cadaver. 1; Distance from nasion, 2; distance from supraorbital margin, 3; distance from infraorbital margin, 4; distance from temporal crest, 5; temporal line, 6; distance from frontal cavity, 7; distance from glabella, 8; distance from midline of face, 9; midline of face, 10; width, 11; distance between frontal notch and supraorbital foramen

### Anatomic Measurements

All measurement was recorded by the same anatomist using a digital caliper (digital vernier calipers of 0.02 mm). Measurements were made and performed in duplicate, and average data were used for increased accuracy and to minimize intra–observer differences. Dry skulls and cadavers that were deformed and whose supraorbital foramen/notch could not be detected were not included in the study. The landmarks of the anatomical measurements we made on cadavers and dry skulls are summarized below.

1. Nasion: It was defined as the point in the midline of both the nasal root and nasofrontal suture [6]. The distance between the supraorbital foramen/notch and the nasion was also measured on the right and left sides in cm (Figure 2.1).
2. Supraorbital margin: The supraorbital margin is the upper edge of the square–shaped opening at the front of the eye socket or the orbital cavity [7]. The distance between supraorbital foramen/notch and the supraorbital margin was measured in cm on the right and left sides (Figure 2.2).
3. Infraorbital margin: The lower edge of the square–shaped opening at the front of the eye socket or the orbital cavity [7]. The distance between supraorbital foramen/notch and the infraorbital margin was measured in cm on the right and left sides (Figure 2.3).
4. Temporal crest: Most anterior and medial point of the inferior temporal line (Figure 2.5), on the zygomatic process of the frontal bone [8]. The distance between the temporal crest on the temporal line and the supraorbital foramen/notch was measured in cm on the right and left sides (Figure 2.4).
5. Frontal cavity: It is the cavity located in the middle of the forehead. The distance between the midline midpoint of the frontal bone and the supraorbital foramen/notch was measured in cm on the right and left sides (Figure 2.6) [9].
6. Glabella: It is present just above the nasion an intersection of internasal and frontonasal suture [10]. The distance between supraorbital foramen/notch and the midpoint of the glabella was measured in cm on the right and left sides (Figure 2.7).
7. Midline of face: The midline of the forehead was established and marked with red pen by connecting trichion to nasion (Figure 2.9) [11]. The distance between the midline of the face and the level where the supraorbital foramen/notch is located was measured in cm on the right and left side (Figure 2.8).

Distance between both foramina/notches: The distance between the right and left side supraorbital foramina/notches was measured bilaterally, yielding values in centimeters.

Distance between frontal foramina/notches and supraorbital foramina/notches: The measurement of the distance between the supraorbital and frontal foramina/notches was taken bilaterally, yielding values in centimeters (Figure 2.11).

Width: The greatest transverse diameters of the supraorbital foramen/notch were recorded on the right and left side (Figure 2.10) at its widest point [12].

#### Statistical Analysis

For comparison of continuous variables between two groups, the Student's t test or Mann–Whitney U test was used depending on whether the statistical hypotheses were fulfilled or not. To evaluate the correlations between measurements, Pearson correlation coefficient or Spearman rank correlation coefficient was used depending on whether the statistical hypotheses were fulfilled or not. Continuous variables were summarized as mean and standard deviation and as minimum–maximum where appropriate. All analyses were performed using IBM SPSS Statistics version 20.0 statistical software package (IBM Corp. Released 2011. IBM SPSS Statistics for Windows, version 20.0 Armonk, NY: IBM Corp). The statistical level of significance for all tests was considered to be 0.05.

## Results

In our study, 56 supraorbital foramen/notch from 28 cadavers and 76 foramen/notch from 38 skulls were examined. Table 1 shows that there were a total of 68 supraorbital foramina and 64 supraorbital notches observed. This indicates that both structures are present in the population, with slightly more foramina than notches. Bilateral occurrences refer to cases where both sides of the supraorbital region have the same structure (either foramina or notches). The table indicates that there were 50 cases of bilateral foramina and 46 cases of bilateral notches. This suggests that bilateral symmetry is common in the distribution of these structures, but foramina are slightly more likely to be bilateral compared to notches. These are instances where one side of the supraorbital region has a different structure from the other side. In this study, there were 36 such cases observed, indicating that asymmetrical configurations are also present in the population, though less common than bilateral symmetry. Moreover, Table 1 breaks down the counts of foramina and notches on the right and left sides separately. For example, there were 36 right–sided foramina and 32 left–sided foramina observed. Similarly, there were 30 right–sided notches and 34 left–sided notches. We also examined the results of the measurements of anatomical parameters on the right and left sides of cadavers (Table 2) and skulls (Table 3).

**Table 1:** Distribution of foramen and notch frequency in cadavers and skulls

Structure	Cadavers (*n*)	Skulls (*n*)	Total (*n*)
Supraorbital foramina	32	36	68
Supraorbital notches	24	40	64
Bilateral foramina	26	24	50
Bilateral notches	18	28	46
Mixed cases	12	24	36
Right foramina	18	18	36
Left foramina	14	18	32
Right notches	10	20	30
Left notches	14	20	34

**Table 2:** Values of the distance of the supraorbital foramen/notch to the parameters in cadavers

Parameters	Cadavers (Right) Mean±SD (Min–Max)	Cadavers (Left) Mean±SD (Min–Max)	Cadavers (Total) Mean±SD (Min–Max)	*p*
Distance from nasion (cm)	2.39±0.46 (1.5–3.4)	2.53±0.48 (1.5–3.5)	2.46±0.47 (1.5–3.5)	0.344
Distance from supraorbital margin (cm)	0.34±0.12 (0.1–0.7)	0.38±0.14 (0.2–0.7)	0.36±0.13 (0.1–0.7)	0.340
Distance from infraorbital margin (cm)	3.38±0.54 (2.1–4.5)	3.45±0.52 (2.6–4.6)	3.41±0.53 (2.1–4.6)	0.645
Distance from temporal crest (cm)	3.79±0.33 (3.1–4.6)	3.58±0.43 (2.7–4.6)	3.69±0.39 (2.7–4.6)	0.042
Distance from midline of face (cm)	2.22±0.49 (1.5–3.3)	2.39±0.57 (1.6–4.3)	2.31±0.53 (1.5–4.3)	0.278
Distance from frontal cavity (cm)	3.28±0.49 (2.3–4.0)	3.29±0.43 (2.5–4.2)	3.29±0.46 (2.3–4.2)	0.850
Distance from glabella (cm)	2.40±0.35 (1.7–3.2)	2.46±0.37 (1.7–3.1)	2.43±0.36 (1.7–3.2)	0.680
Width (cm)	0.38±0.09 (0.2–0.5)	0.39±0.13 (0.1–0.7)	0.39±0.11 (0.1–07)	0.891

SD, Standard Deviation: Min, Minimum: Max, Maximum: *p*, Independent Samples Mann– Whitney *U* test values

**Table 3:** Values of the distance of the supraorbital foramen/notch to the parameters in skulls

Parameters	Skulls (Right) Mean±SD (Min–Max)	Skulls (Left) Mean±SD (Min–Max)	Skulls (Total) Mean±SD (Min–Max)	*p*
Distance from nasion	2.32±0.54 (1.6–4.0)	2.40±0.49 (1.8–3.7)	2.36±0.52 (1.6–4.0)	0.346
Distance from supraorbital margin	0.37±0.15 (0.2–0.7)	0.36±0.12 (0.2–0.6)	0.37±0.13 (0.2–0.7)	0.932
Distance from infraorbital margin	3.58±0.32 (2.7–4.2)	3.66±0.35 (2.6–4.3)	3.62±0.34 (2.6–4.3)	0.256
Distance from temporal crest	3.14±0.61 (1.5–4.2)	3.01±0.52 (1.6–4.0)	3.07±0.56 (1.5–4.2)	0.203
Distance from midline of face	2.12±0.49 (1.5–3.5)	2.17±0.47 (1.5–3.3)	2.14±0.48 (1.5–3.5)	0.673
Distance from frontal cavity	2.66±0.48 (1.8–4.2)	2.69±0.45 (1.9–3.5)	2.68±0.46 (1.8–4.2)	0.673
Distance from glabella	2.20±0.51 (1.4–3.7)	2.21±0.46 (1.6–3.2)	2.20±0.49 (1.4–3.7)	0.967
Width	0.43±0.12 (0.2–0.7)	0.42±0.13 (0.2–0.6)	0.43±0.13 (0.2–0.7)	0.797

SD, Standard Deviation: Min, Minimum: Max, Maximum: *p*, Independent Samples Mann–Whitney *U* test values

**Fig. 3:**
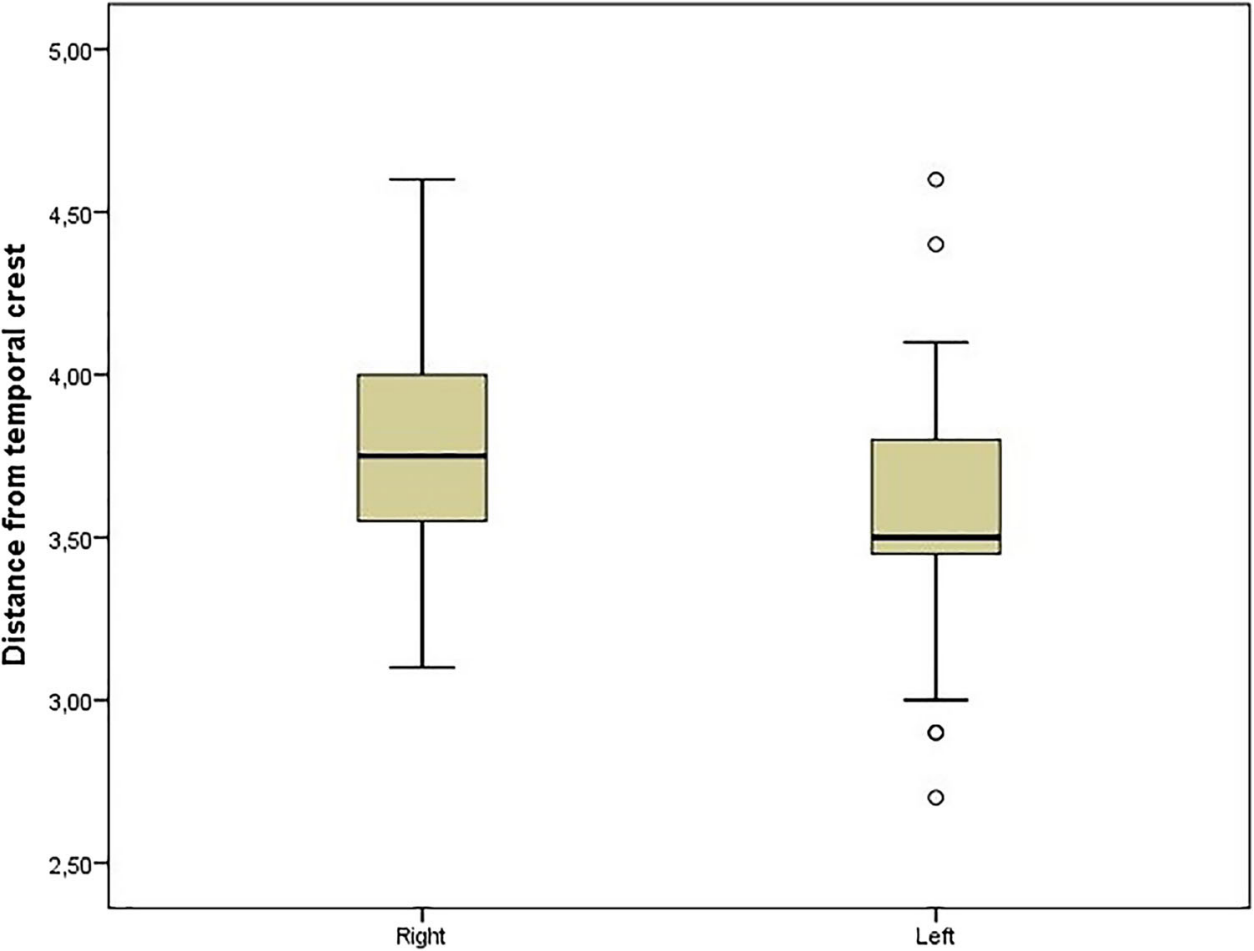
Significant result of cadaver measurements between right and left sides distributions of parameters

**Fig. 4:**
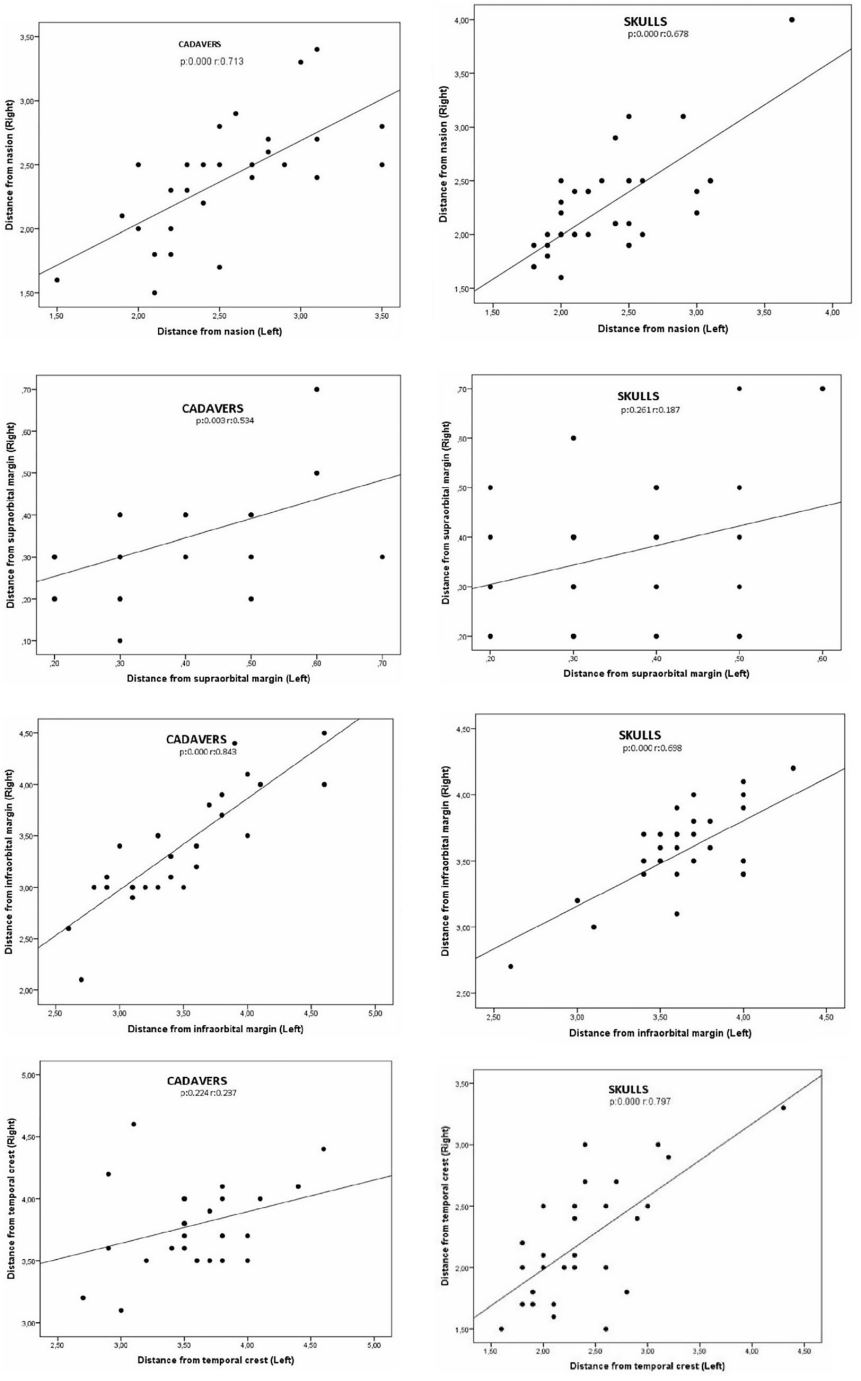
Right and left sides correlations of parameters in cadavers and skulls; part 1

**Fig. 5:**
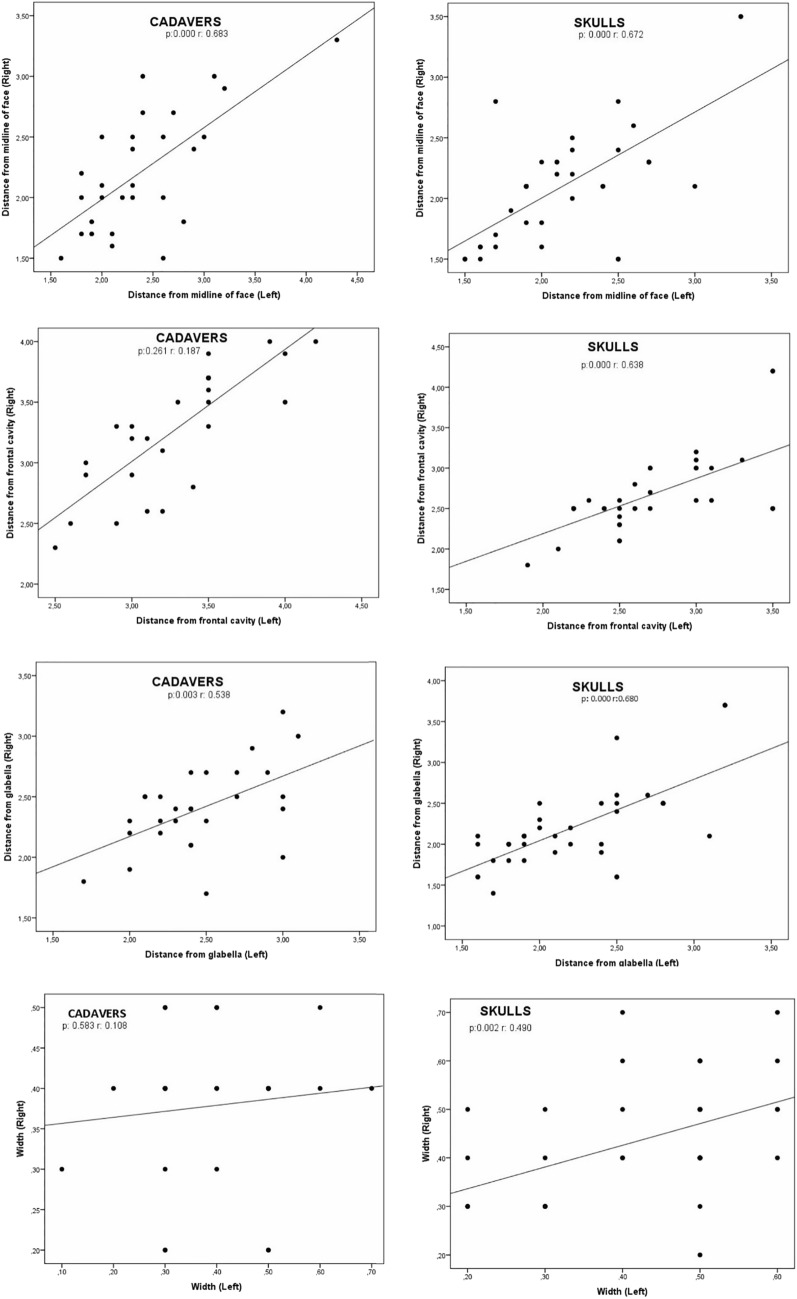
Right and left sides correlations of parameters in cadavers and skulls; part 2

In our study, similarity was found in the right and left sides dimension and location values of the anatomical measurement parameters in cadaver and dry skulls, except for the "distance from temporal crest" parameter (Table 2, Figure 3). A significant difference was obtained between the right and left sides of the cadavers in the "distance from temporal crest" parameter (*p*=0.042) (Table 2). Additionally, in the correlation analysis, a positive significant relationship was found between the right and left sides in all parameters except "distance from supraorbital margin" (*r*: 0.187, *p*: 0.261) in dry skull, "distance from temporal crest" (*r*: 0.237, *p*: 0.224), "distance from frontal cavity" (*r*: 0.187, *p*: 0.261) and "width" (*r*: 0.108, *p*: 0.583) in cadaver parameters (Figures 4 and 5). Moreover, the average distance between both foramina was found to be 4.68±0.69 cm in cadavers and 4.46±0.80 cm in dry skulls. While no significant difference was obtained between the cadaver and skull in the distance between frontal notch and supraorbital foramen/notch, a significant difference was obtained in the distance between frontal foramen and supraorbital foramen/notch (Table 4).

**Table 4:** Values of the distance between foramen/notch frontale and foramen/notch supraorbitale

	Cadavers Mean±SD (Min–Max)	Skulls Mean±SD (Min–Max)	Total Mean±SD (Min–Max)	*p*
Distance from frontal notch	0.96±0.49 (0.2–1.6)	0.83±0.41 (0.3–1.3)	0.95±0.46 (0.2–1.6)	0.546
Distance from frontal foramen	0.98±0.42 (0.3–1.5)	0.51±0.21 (0.3–0.9)	0.68±0.37 (0.2–1.5)	0.017

SD, Standard Deviation: Min, Minimum: Max, Maximum: *p*, Independent Samples Mann–Whitney *U* test values

## Discussion

The glabella and supraorbital region represent critical anatomical areas targeted in cosmetic procedures, albeit with inherent risks of complications. Literature reports document a spectrum of serious or permanent complications associated with these procedures, with a notable focus on the supraorbital arteries and their intricate course through the orbital bone. Complications such as retrograde filler flow into the ophthalmic artery and resultant skin ischemia underscore the importance of meticulous technique and anatomical knowledge in such interventions. Injectable substances like calcium hydroxyapatite and hyaluronic acid are frequently employed in these procedures, necessitating precise injection techniques to minimize risks. Additionally, accurate localization of anatomical landmarks, particularly the supraorbital foramen/notch, assumes paramount importance in ensuring procedural safety and optimizing outcomes [13–15]. The complexity of the glabella and supraorbital region anatomy presents unique challenges in cosmetic procedures, necessitating a thorough understanding of anatomical variations and associated risks. Strategies to mitigate complications, including precise injection techniques and awareness of danger zones, are essential for ensuring procedural safety. Variations in supraorbital foramen/notch anatomy, often observed in patients with primary trigeminal neuralgia, highlight the need for meticulous pre–procedural assessment to minimize risks. Furthermore, the implications of anatomical variations extend beyond aesthetic procedures, with potential diagnostic significance in identifying underlying pathologies. Overall, a comprehensive understanding of the supraorbital anatomy and meticulous attention to detail is paramount in optimizing outcomes and minimizing complications in cosmetic procedures targeting the glabella and supraorbital region [16–18].

**Fig. 6:**
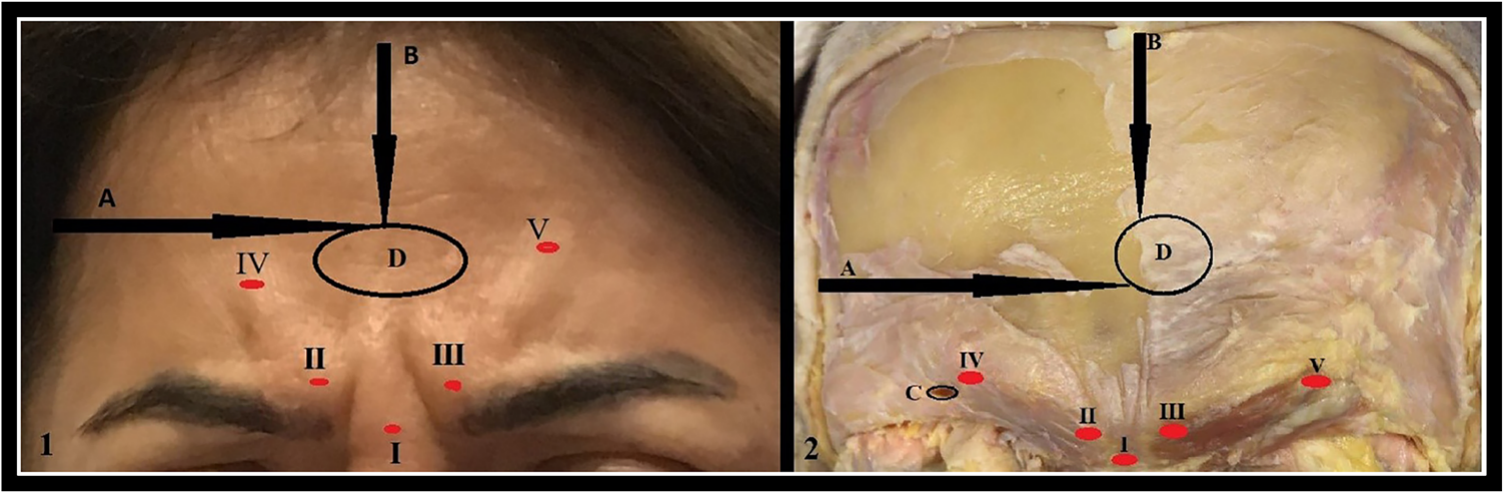
Illustration of approximate reference points of some medical aesthetic applications. 1; living individual, 2; cadaver, I–II–III–IV–V points; botulinum toxin application points on the glabella area, A–B; recommended cannula entry direction for medical aesthetic applications, D; frontal cavity

**Fig. 7:**
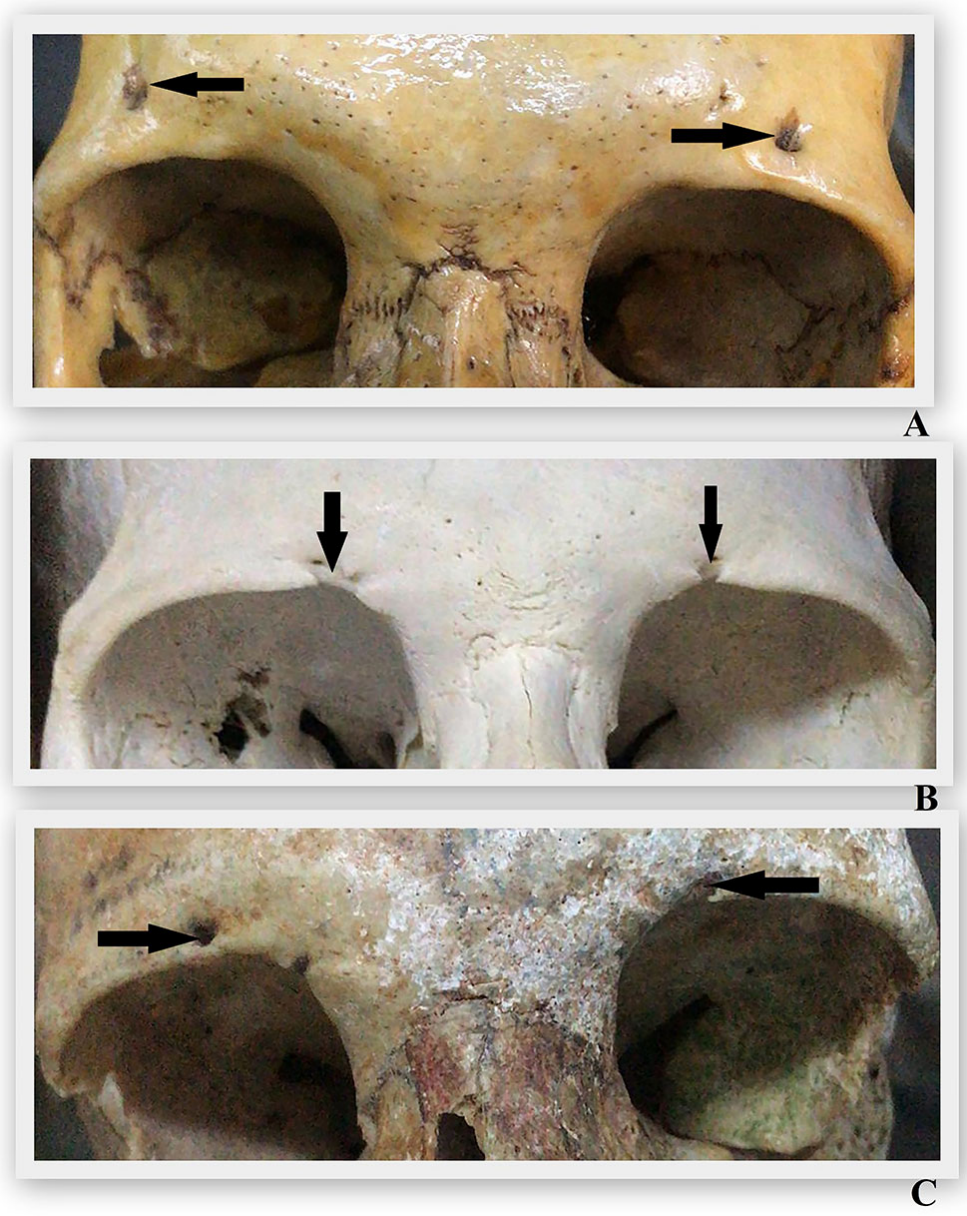
Examples of supraorbital foramen and supraorbital notch on skulls (**A**; supraorbital foramen bilaterally, **B**; supraorbital notch bilaterally, **C**; supraorbital foramen on right side and supraorbital notch on left side)

For optimal safety and precision in cosmetic procedures targeting the glabella and frontal areas, it is recommended to employ superficial injections utilizing either a cannula bolus or fan technique at specified locations, as indicated by the "A and B" arrows in Figure 6. In our investigation, meticulous measurements were taken to assess the distance between the temporal crest along the temporal line and the frontal cavity centrally situated within the frontal bone. Notably, particular attention was directed toward evaluating the arcus supercilia and the supraorbital foramen/notch. A profound comprehension of the complex anatomical structures comprising the supraorbital area and orbital roof is imperative to avert inadvertent injections into regions identified as "danger zones," thus mitigating the associated risk of eyelid ptosis [19]. Furthermore, a common practice in cosmetic procedures involves the administration of botulinum toxin at the midpoint of the eyebrow. Anatomical landmarks, denoted as Points I, II, III, IV and V in Figure 6, serve as pivotal reference points for such injections, particularly in the glabella region. However, it is crucial to recognize the potential hazards posed by these injection sites to the supraorbital foramen/notch and its adjacent structures. Consequently, our study included meticulous evaluation parameters, which encompassed the measurement of distances between the supraorbital foramen/notch and key facial landmarks, such as the glabella, nasion and midline of the face. These anatomical landmarks play a pivotal role as they provide valuable insights into the precise origins (foramen supraorbitale or notch supraorbitale) of the arterial, venous and supraorbital nerve structures, thereby aiding in the formulation of safe and effective injection strategies. Thus, these findings in this study provide quantitative insights into the spatial relationships between key anatomical landmarks and the injection sites, contributing to the understanding of safe and precise injection techniques in cosmetic procedures targeting the glabella and frontal areas (Fig. 7).

**Fig. 8:**
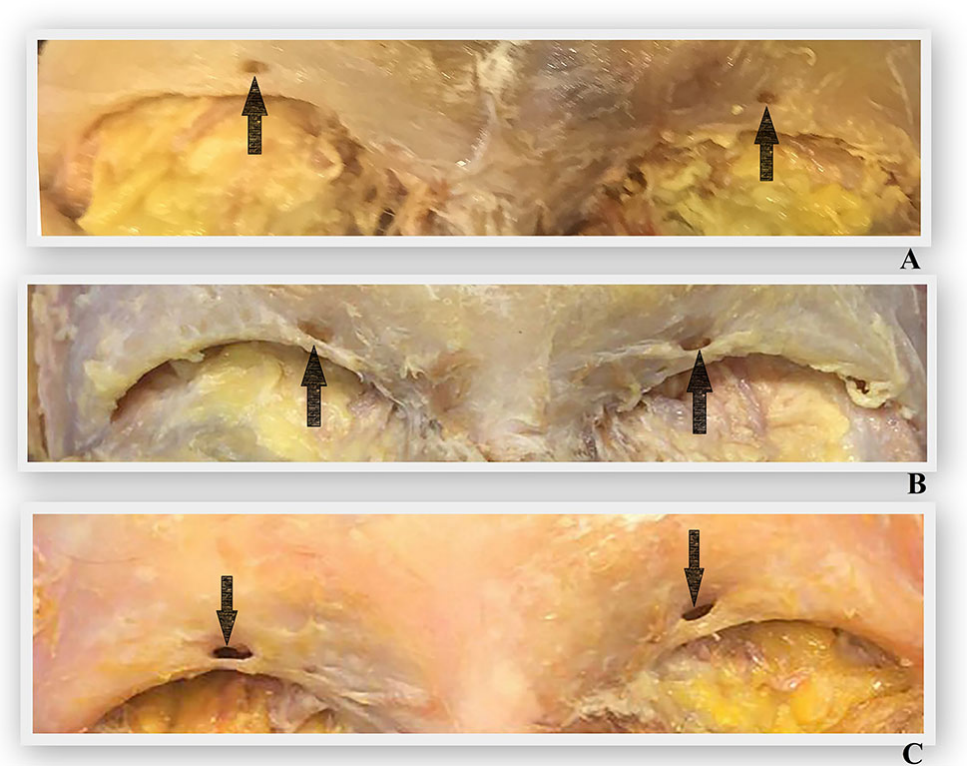
Examples of supraorbital foramen and supraorbital notch on cadavers (**A**; supraorbital foramen bilaterally, **B**; supraorbital notch bilaterally, **C**; supraorbital foramen on left side and supraorbital notch on right side)

In our study, we examined a total of 132 supraorbital foramen/notch structures from 28 cadavers and 38 skulls. Of these, 68 (32 in cadavers, 36 in skulls) were supraorbital foramina, and the remaining 64 (24 in cadavers, 40 in skulls) were supraorbital notches. In addition, in our study, bilateral supraorbital foramina (Figure 7A, Figure 8A) and bilateral supraorbital notch (Figure 7B, Figure 8B) were recorded, while asymmetric formations were also recorded (Figure 7C, Figure 8C). By providing counts for both cadavers and skulls separately, Table 1 allows for comparisons between these two groups. For instance, it shows that there were slightly more supraorbital foramina observed in skulls compared to cadavers (36 vs. 32), while the opposite trend is observed for supraorbital notches (40 vs. 24). This could be due to differences in preservation or other factors related to the study sample. Overall, results of our study provide valuable insights into the prevalence and distribution of supraorbital foramina and notches in the study population, which can contribute to our understanding of anatomical variations and potentially have implications for clinical practice or forensic identification. Furthermore, the following results were found: In both cadavers and skulls, there is no significant difference in the distance from nasion between the right and left sides. Similarly, the distance from the supraorbital margin shows no significant difference between the right and left sides in both cadavers and skulls. Also, there is no significant difference between the right and left sides in skulls distance foramen infraorbital margin, but in cadavers, this difference is not stated. In addition, cadavers show a significant difference between the right and left sides distance from temporal crest, while skulls do not exhibit a significant difference. Neither cadavers nor skulls show a significant difference distance from midline of face, distance from frontal cavity, distance from glabella and width on the right and left sides. Overall, there is no significant difference between the measurements of cadavers and skulls in most parameters. This suggests that the anatomical features measured are relatively consistent between these two groups, indicating the utility of skulls as proxies for cadavers in certain studies or analyses. Ultimately, the results of our study provide valuable information about anatomical measurements in cadavers and skulls, as well as the symmetry of these measurements between the right and left sides. This information can be useful in various fields such as anthropology, anatomy and forensic science. Moreover, while a significant difference was found between the distance from the foramen frontale and cadaveric specimens versus skulls, no significant difference was observed concerning the distance from the frontal notch. This discrepancy may be attributed to the detection of a total of 3 frontal foramina, as mentioned in the literature where the frequency of frontal notches is high. The measurement of the distance between the frontal foramen/notch and the supraorbital foramen/notch holds significant clinical importance in various contexts. Precise anatomical knowledge in surgical and cosmetic procedures is crucial to avoid injury to neurovascular structures. Additionally, understanding anatomical variations informs personalized treatment approaches. Moreover, accurate localization for nerve block anesthesia ensures optimal patient comfort during procedures. It guides surgical and cosmetic procedures, accommodates anatomical variations and ultimately ensures optimal patient safety and treatment outcomes.

Studies in the literature have indeed explored similar parameters using various methodologies, including cadaveric dissections, examination of skulls and analysis of CT images. The literature presents variations in the prevalence of supraorbital structures across different populations. For example, the study in Bosnia and Herzegovina found bilateral supraorbital notches in 58.33% of cases [1], whereas in Thai skulls, the supraorbital foramen was more prevalent, being present in a notch form in only 13.8% of cases [5]. Additionally, Hong et al. observed a higher prevalence of the supraorbital notch compared to the foramen in their study utilizing 3–dimensional facial bone CT scans  [2]. This finding aligns with the prevalence observed in the Thai population. These variations in prevalence highlight the importance of considering ethnic differences when analyzing supraorbital anatomy and planning surgical interventions.

Moreover, the studies report morphometric measurements such as width, distances from various landmarks and the presence of bilateral or unilateral structures. The literature provides insights into the mean width of the supraorbital foramen/notch, distances from landmarks like the midline of the face and temporal crest, and variations in these measurements between different populations. For example, the study by Hester et al. on cadavers reported a mean distance between the midline of the face and the supraorbital foramen/notch of 25.3 ± 3.9 mm. This measurement varied significantly based on ethnic origins, as noted in their analysis of European and Hispanic cadavers [3]. Also, the prevalence of bilateral and unilateral supraorbital structures varies across populations. For instance, the study on Sri Lankan skulls found that 55.56% of individuals exhibited bilateral supraorbital notches, while 20.37% had bilateral supraorbital foramina [10]. Furthermore, a significant percentage of cases displayed a notch on one side and a foramen on the contralateral side, emphasizing the asymmetry commonly observed in the supraorbital region. Understanding the variations in supraorbital anatomy and morphometric characteristics is crucial for various clinical practices, including surgical interventions, facial reconstruction and forensic identification. Ethnic variations in supraorbital anatomy, as highlighted in these studies, underscore the importance of personalized approaches in clinical practice, considering the specific characteristics of each population. In summary, integrating the findings from the literature with the provided results in this study enhances our understanding of supraorbital anatomy, prevalence and morphometric characteristics across different populations, providing valuable insights for clinical and research purposes.

The primary limitation of our study is the lack of recorded measurements of all cadavers and skulls studied, irrespective of sex and ethnicity. While our study primarily focused on anatomical measurements related to the supraorbital foramen/notch and surrounding structures, we acknowledge certain limitations. We did not specifically measure the supraorbital nerve branches, including its inferior branch, nor did we assess the incidence of the existence of this branch as part of our study objectives. However, given the limitations of our study, we were unable to provide data on this specific aspect. Moreover, we did not specifically measure the distance between the corrugator supercilii muscle and the supraorbital foramen/notch in our study. We recommend conducting current studies that investigate the relationship between the vascular and neural structures within the supraorbital foramen or notch and the adjacent muscles.

## Conclusion

These findings suggest consistency in supraorbital foramen/notch anatomy across our study sample and support the reliability of our results when compared to existing literature. These results contribute to the existing literature on supraorbital anatomy and variations, enhancing our understanding of this region's characteristics.

## References

[1] Voljevica A, Talović E, Šahinović M et al (2022) Morphometric analysis of the supraorbital foramen and notch in the population of Bosnia and Herzegovina. Acta Med Acad 51(2):92–98. 10.5644/ama2006-124.377 36318001 10.5644/ama2006-124.377 PMC9982859

[2] Hong JH, Kim JS, Shin HK (2021) Studies of supraorbital notch and foramen using 3–dimensional facial bone ct scans. Pain Physician 24(8):E1273–E1278 34793654

[3] Hester KM, Rahimi OB, Fry CL et al (2021) The relative locations of the supraorbital, infraorbital, and mental foramina: a cadaveric study. J Anat 239(4):782–787. 10.1111/joa.13482 34120334 10.1111/joa.13482 PMC8450472

[4] Turhan-Haktanir N, Ayçiçek A, Haktanir A et al (2008) Variations of supraorbital foramina in living subjects evaluated with multidetector computed tomography. Head Neck 30(9):1211–1215. 10.1002/hed.20866 18642294 10.1002/hed.20866

[5] Chao YYY (2018) Saline hydrodissection: a novel technique for the ınjection of calcium hydroxylapatite fillers in the forehead. Dermatol Surg 44(1):133–136. 10.1097/DSS.0000000000001136 28445197 10.1097/DSS.0000000000001136

[6] Bidra AS, Uribe F, Taylor TD et al (2009) The relationship of facial anatomic landmarks with midlines of the face and mouth. J Prosthet Dent 102(2):94–103. 10.1016/S0022-3913(09)60117-7 19643223 10.1016/S0022-3913(09)60117-7

[7] Thunyacharoen S, Singsuwan P, Mahakkanukrauh P (2022) Morphometric studies of supraorbital foramen, infraorbital foramen and mental foramen in a Thai population related with nerve blocks. Int J Morphol 40(1):181–187. 10.4067/s0717-95022022000100181

[8] Bermejo E, Taniguchi K, Ogawa Y et al (2021) Automatic landmark annotation in 3D surface scans of skulls: methodological proposal and reliability study. Comput Methods Programs Biomed 210:106380. 10.1016/j.cmpb.2021.106380 34478914 10.1016/j.cmpb.2021.106380

[9] Van Loghem JAJ (2018) Use of calcium hydroxylapatite in the upper third of the face: retrospective analysis of techniques, dilutions and adverse events. J Cosmet Dermatol 17(6):1025–1030. 10.1111/jocd.12733 30362225 10.1111/jocd.12733

[10] Walker HM, Chauhan PR (2024) Anatomy, head and neck: glabella. In: StatPearls [Internet]. Treasure Island (FL): StatPearls Publishing 32310453

[11] Pruksapong C, Kawichai W, Attainsee A et al (2022) The anatomical variations of the emergence routes of supraorbital nerve: a cadaveric study and systematic review. Asian J Surg 45(1):220–225. 10.1016/j.asjsur.2021.04.048 34167870 10.1016/j.asjsur.2021.04.048

[12] Ilayperuma I, Nanayakkara G, Palahapitiya N (2014) Supraorbital notch/foramen in Sri Lankan skulls: morphometry and surgical relevance. Int JMorphol 32:435–439

[13] Oranges CM, Brucato D, Schaefer DJ et al (2021) Complications of nonpermanent facial fillers: a systematic review. Plast Reconstr Surg Glob Open 9(10):e3851. 10.1097/GOX.0000000000003851 34703713 10.1097/GOX.0000000000003851 PMC8542164

[14] Scheuer JF, Sieber DA, Pezeshk RA et al (2017) Anatomy of the facial danger zones: maximizing safety during soft–tissue filler injections. Plast Reconstr Surg 139:50e–58e 28027232 10.1097/PRS.0000000000002913

[15] Rohrich RJ, Bartlett EL, Dayan E et al (2019) Practical approach and safety of hyaluronic acid fillers. Plast Reconstr Surg Glob Open 7:e2172 31624663 10.1097/GOX.0000000000002172 PMC6635180

[16] Minelli L, Richa J, Mendelson BC (2022) Aesthetic enhancement of the brow using hydroxyapatite. Aesthetic Plast Surg 46(3):1201–1210. 10.1007/s00266-022-02793-y 35288761 10.1007/s00266-022-02793-y PMC9411237

[17] Xie K, Liu S, Huang B et al (2020) Effects of supraorbital foramen variations on the treatment efficacy of radiofrequency therapy for v1 trigeminal neuralgia: a retrospective study. Pain Res Manag 26(2020):8142489. 10.1155/2020/8142489 10.1155/2020/8142489 PMC7061117 32184911

[18] Tansatit T, Phumyoo T, Jitaree B et al (2020) Anatomical and ultrasound–based injections for sunken upper eyelid correction. J Cosmet Dermatol 19(2):346–352. 10.1111/jocd.13049 31222959 10.1111/jocd.13049

[19] Nestor MS, Han H, Gade A et al (2021) Botulinum toxin–induced blepharoptosis: anatomy, etiology, prevention, and therapeutic options. J Cosmet Dermatol 20(10):3133–3146. 10.1111/jocd.14361 34378298 10.1111/jocd.14361 PMC9290925

